# A Case of Suppurative Pleuritis with Gangrene of a Portion of Lung Tissue and Its Escape from the Operation Wound

**Published:** 1881-03

**Authors:** W. Wagner, P. W. Van Peyma

**Affiliations:** Koenigshuette


### A CASE OF SUPPURATIVE PLEURITIS WITH GANGRENE OF A PORTION OF LUNG TISSUE AND ITS ESCAPE FROM THE OPERATION WOUND.—BY DR. W. WAGNER, KOENIGSHUETTE.

FROM THE GERMAN BY P. W. VAN PEYMA, M. D.

"PUTRIDE PLEURITIS" is a disease, which has only in more recent times received accurate study. Being at the same time comparatively rare, every case observed would appear to be worthy of publication. But the case in point possesses much additional interest in that it was accompanied by the loss through the operation wound of a mass of gangrenous lung tissue 7 centimeters in length. Similar cases have very rarely been observed, at least my knowledge of the literature of the subject does not include any.

J. Wosny, mountaineer, 17 years of age, was admitted to the hospital on the 14th of October, 1878. He suffered from dysentery of moderate severity, which was treated by irrigation of the bowels with salicylic water and which, during its course, presented no points of particular interest.

On the 6th of November, three weeks after admission, the patient having been convalescent for a week, he was suddenly attacked with a chill and pain in the left side. An examination showed a well developed pneumonia of the left lower lobe, with dullness, bronchial respiration, increased vocal fremitus, and with an occasional friction murmur in the axillary region. Expectoration glairy, intermixed with bright blood; temp. 41 C.; pulse 120, small and hard. General condition, as regards vitality, unfavorable, even on the first day.

The temperature, which had since the cold baths been under 40, fell, on the eighth day of the sickness, to 38.5. It continued at about this point for a number of days, and then gradually dropped to normal on the twenty-first. During the lysis the dullness gradually disappeared; subcrepitant rales were heard with weak vesicular breathing. The sputum became simple purulent and diminished in quantity; in short, the case was one of regular pneumonia, ending in lysis.

After the patient had been for three days entirely free from fever, he was again attacked on the 24th with severe pain in the left side and the temperature rose to 39.5. That the patient was during the three preceding days entirely free from fever, is an important point and is positively established, as thermometric examination was made every three hours.

The examination made early on the 25th, showed flatness in the neighborhood of the apex of the left scapula, extending to the left axillary region; the respiratory murmur was nearly entirely absent, in parts a very distant bronchial respiration. Vocal fremitus was in this region entirely suspended.

The diagnosis of acute suppurative pleuritis was clear.

On the 27th the effusion had reached to the middle of the scapula; above the region of dullness small mucous rales were heard with complementary respiration. The heart was crowded strongly to the right. An exploratory puncture resulted in the escape of a quantity of whitish pus, of slight consistence and of a disagreeable odor. The microscopical examination showed in addition to masses of pus corpuscles a large number of bacteria.

With antiseptic precautions an opening for exit was made in the eighth intercostal space and about 700 grammes of the above mentioned pus was evacuated. The odor, while disagreeable, could hardly be termed putrid. After irrigation with a three-per cent. solution of carbolic acid, a thick drain was introduced, this covered with Lister's dressing, salicylated cotton and carbolized jute. The dressing was necessarily removed twice daily. The pus became thinner and more and more disagreeable in odor until the 2d of December, when it had become strongly fetid. The temperature constantly in the neighborhood of 40, the pulse proportionally frequent; general condition bad.

On the day last mentioned, as the dressings were being renewed, a brownish mass presented itself at the wound, and partly protruded. By means of forceps a slight traction was made, and a mass of gangrenous lung, seven cent. long by three cent. wide and thick, popped out. Attached to this mass were shreds of pleural membrane. The odor was very fetid.

The microscopical examination made by Prof. Ponfik showed gangrenous lung tissue, in which the hepatization was very apparent. For a number of days the washing out with three per cent. carbolic acid solution was continued, and the odor gradually improved. The secretion was nevertheless quite considerable, the temperature was now but moderately increased, the general condition improved.

By the 15th of January, 1879, the secretion had become slight, and the general condition very good. At this time the temperature again rose to 40°; the scanty discharge became fetid, and the patient began to expectorate purulent matter. The drain which had for some time been quite short, could on the twenty-fifth no longer be introduced, and I began to fear retention of pus. By means of the careful introduction of an elastic catheter, a teacupful of thickish and noisome pus escaped.

The granulations which obstructed the opening were removed by means of the sharp spoon, and in consideration of the offensive odor, the pleural cavity was washed out with a three per cent. solution of carbolic acid water. During this irrigation the patient was suddenly affected with coughing and nausea, and a quantity of the carbolic acid water mixed wth mucous and pus was evacuated by the mouth.

As an experiment, colored liquids were injected into the cavity, and these were likewise evacuated by the mouth. A two per cent. carbolic acid solution was now inhaled.

By Feb. 7 the offensive odor of the pus as well as of the expectoration had entirely disappeared; the secretion was scanty and the quantity of expectoration much diminished. Temperature normal; general condition good. The fluids introduced into the pleural cavity are still ejected by the mouth.

On Feb. 16, I demonstrated this experiment before the Medical Society. The drain probably fell out on the way home, as upon removing the dressing afterwards the wound was found to have closed, and it, from that time on, remained so. The expectoration was still yellowish and contained a moderate amount of pus corpuscles.

Eight days after the closure of the wound the physical condition of the thorax was as follows: moderate dullness over the lower part of the axillary and infra scapular regions; enfeebled vesicular murmur and vocal fremitus and quite numerous subcrepitant rales. The expansion of the left side of the thorax, as compared with the right, was much diminished.

On July 6th the patient returned to his hard work, as a mountaineer, healthy and rugged. The left side of the thorax was now but little inferior to the right, as regards expansion. Only in the vicinity of the cicatrix were there heard a few rales. In the lower part of the left axillary region there was slight dullness and somewhat enfeebled respiratory murmur. The cardiac impulse had returned to the left fifth costal interspace, two centimeters outside the line of the nipple.

The article concludes with remarks by the writer.—*Berliner Klinische Wochenschrift.*

